# Polymer Micro and Nanoparticles Containing B(III) Compounds as Emissive Soft Materials for Cargo Encapsulation and Temperature-Dependent Applications

**DOI:** 10.3390/nano11123437

**Published:** 2021-12-18

**Authors:** Frederico Duarte, Cristián Cuerva, Carlos Fernández-Lodeiro, Javier Fernández-Lodeiro, Raquel Jiménez, Mercedes Cano, Carlos Lodeiro

**Affiliations:** 1 BIOSCOPE Research Group, LAQV@REQUIMTE Chemistry Department, NOVA School of Science and Technology, NOVA University Lisbon, 2829-516 Caparica, Portugal; fg.duarte@campus.fct.unl.pt (F.D.); j.lodeiro@fct.unl.pt (J.F.-L.); 2 MatMoPol Research Group, Department of Inorganic Chemistry, Complutense University of Madrid, Ciudad Universitaria, 28040 Madrid, Spain; raquel1580@hotmail.com (R.J.); mmcano@ucm.es (M.C.); 3 CINBIO, Departamento de Química Física, Campus Universitario Lagoas Marcosende, Universidade de Vigo, 36310 Vigo, Spain; carfernandez@uvigo.es; 4 Galicia Sur Health Research Institute (IIS Galicia Sur), SERGAS-UVIGO, 36310 Vigo, Spain; 5 PROTEOMASS Scientific Society, Rua dos Inventores, Madam Parque, Caparica Campus, 2829-516 Caparica, Portugal

**Keywords:** organoboranes, polymer nanoparticles, drug delivery, temperature sensors

## Abstract

Polymer nanoparticles doped with fluorescent molecules are widely applied for biological assays, local temperature measurements, and other bioimaging applications, overcoming several critical drawbacks, such as dye toxicity, increased water solubility, and allowing imaging of dyes/drug delivery in water. In this work, some polymethylmethacrylate (PMMA), polyvinylpyrrolidone (PVP) and poly(styrene-butadiene-styrene) (SBS) based micro and nanoparticles with an average size of about 200 nm and encapsulating B(III) compounds have been prepared via the reprecipitation method by using tetrahydrofuran as the oil phase and water. The compounds are highly hydrophobic, but their encapsulation into a polymer matrix allows obtaining stable colloidal dispersions in water (3.39 µM) that maintain the photophysical behavior of these dyes. Although thermally activated non-radiative processes occur by increasing temperature from 25 to 80 °C, the colloidal suspension of the B(III) particles continues to emit greenish light (λ = 509 nm) at high temperatures. When samples are cooling back to room temperature, the emission is restored, being reversible. A probe of concept drug delivery study was conducted using coumarin 6 as a prototype of a hydrophobic drug.

## 1. Introduction

In vivo fluorescent imaging studies, control of drug delivery success and local temperature measurements in biological and medicinal systems are critical tasks [1,2,3,4]. The results are unambiguous when the emissive molecular organic dye or fluorescent metal complex is poorly soluble in water. To overcome these problems, dye-doped polymer nanoparticles can be used to prevent poor solubility, reduce the interference with the assay, control the pH-dependent analysis, prevent unexpected quenching, low absorptivity, and often maintain photostability in imaging and drug release applications [5,6,7,8].

During recent years different dye-doped polymer nanoparticles have emerged as biocompatible materials with a wide range of particle size from nano to micro-scale, minimizing the interference between the dye and the assay that is accomplished by immobilization; controlling concentration to induce increased fluorescence and absorbance properties, and also protecting the fluorescent molecule against oxidation or any enzymatic degradation [9,10]. One example of the usefulness of entrapping dyes into polymer nanoparticles was reported by J. Desbiens et al. Several polystyrene (PS) nanoparticles were synthesized and doped with a luminescent europium complex, Eu(tta)_3_phen, spanning in size from 19 to 94 nm. These nanomaterials were dispersed in water through a mini-emulsion polymerization approach that allowed the photophysical properties of the dye to remain unaltered, as observed in THF solution even though this compound is insoluble in water [11]. By contrast, M. Amela-Cortes et al. have demonstrated the homogeneity, stability, and the maintenance of the luminescent properties of poly(methylmethacrylate) (PMMA) nanocomposites doped with different hexanuclear octahedral clusters via free-radical polymerization [12].

In this field, N. Visaveliya et al. reported several synthetic approaches of fluorescent polymer nanoparticles using a micro-flow assisted technique in which an aqueous phase interacts with the monomer phase in a crossflow arrangement of the microreactor. The aqueous glass syringe contains either cetyltrimethylammonium bromide (CTAB) or sodium dodecyl sulfate (SDS) as stabilizers, and the monomer syringe contains ethylene glycol dimethacrylate (EGDMA) cross-linker, thermal initiator azobisisobutyronitrile (AIBN) and methyl methacrylate (MMA), which are essential for the polymerization of the corresponding PMMA nanoparticles [10].

On the same note, X. Zhang et al. described the latest advances of different strategies for the development of polymer luminescent nanomaterials resorting to dyes that manifest aggregation-induced emission (AIE) properties and how it can cause a significant impact in several research fields, making its way through chemistry until biology fields and, obviously, the preeminent research field of materials and all interdisciplinary areas between them. For this reason, the authors highlighted various strategies on how to design a multitude of nanomaterials using this sort of dyes by self-assembly, the ability to create new covalent bonds between the dye and the polymer matrix, and some polymerization techniques, namely ring-opening and free radical polymerization among other methodologies. Finally, a position is taken on how these materials can be applied to bioimaging, biosensors and, as nano theragnostic systems; however, there is still yet the implication for developing more sophisticated new systems not only to provide fluorescence image but to fulfil the modern requirements that nanomedicine may come across [13].

Taking into consideration this idea, extensive studies were conducted regarding the photoluminescence properties applied to the design of a broad spectrum of nanomaterials and their implications in the research fields of chemistry and biology through imaging and analysis [14]. A subsequent and new approach has arisen from this early conceptualization of nanotechnology and the cornerstone of research fields, giving them a fresh perspective. For this reason, in recent years, nanomedicine has marked its way through and set its roots as a promising strategy to improve drug-delivery methodologies to diagnose and treat a vast number of diseases and cancer. It is well known that this approach can overcome diverse challenges that conventional techniques face by creating new strategies that make possible the reduction of toxicity and side effects, translating into nanocarriers capable of improving the bioavailability and administration of new drugs as well as to monitoring in space and time to provide more control and efficiency [15,16].

For example, Y. Fan et al. discuss the fabrication of CdSeS/ZnS quantum dots entrapped in the co-polymer poly (methyl methacrylate-co-methacrylic acid) (PMMA-co-MAA) through precipitation and assembly of these compounds, initially dissolved in a THF solution, upon addition to an aqueous solution. These materials were found to be highly photostable, with low levels of cytotoxicity and high colloidal stability [17].

Meanwhile, diruthenium metallodrugs containing ibuprofen and naproxen have been reported by S. Rico et al. and encapsulated into injectable solid polymer-lipid nanoparticles, which demonstrated to be cytotoxic to the breast (EMT6 and MDA-MB-231) and prostate (DU145) cancer cells in vitro [18].

By a less complicated methodology, covalently binding doped polylactic acid-polyethylene glycol (PLA-PEG) nanoparticles have been synthesized by C. Kerr et al. connecting difluoroboron β-diketonate poly(lactic acid) particles as a biological imaging agent. The dye loading and tumor accumulation was confirmed using a biodistributed model [19].

Although polymer nanoparticles can provide many benefits to the diverse research fields, it is also essential to look out for their adverse impact, especially on the environment. For this reason, Y. J. Yip et al. have investigated the toxicity level of three different common types of polymer nanoparticles such as PMMA, PS, and polyvinylchloride (PVC) doped with perylenetetraester dye on acorn barnacle (*amphibalanus amphitrite*). Results suggested that both PMMA and PS nanoparticles did not show significant toxicity and that mortality levels were insufficient to obtain LC_50_ value. The opposite results were obtained for PVC nanoparticles since 99% of the population would die in contact with these nanomaterials at a concentration of 25 mg/L [20].

**Figure 1:**
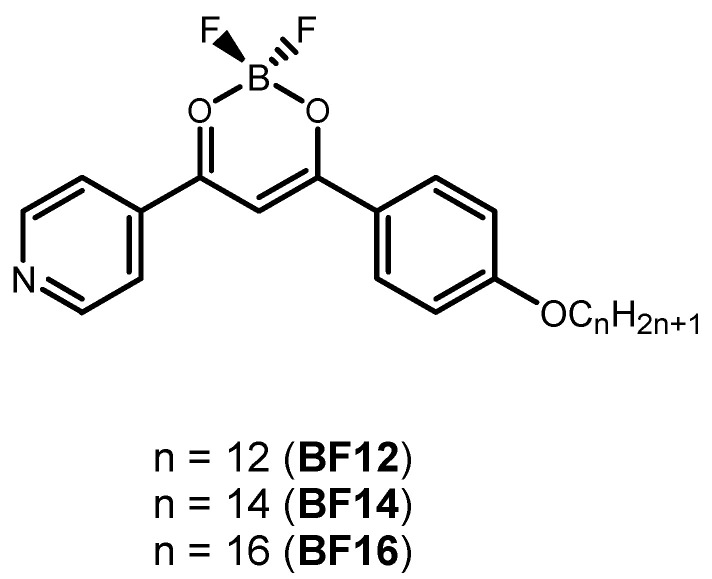
Molecular structure of the B(III) compounds previously synthesized by us [22].

Based on our previous expertise in synthesizing and studying B(III) compounds used as solid-state and solution emitters [21,22], we have used a parallel methodology to entrap these dyes into polymer micro and nanostructures using a green methodology that allows reducing waste materials [23,24,25]. The compounds selected for these studies are shown in Figure 1. Dispersion into polymer particles is used as a strategy to avoid the aggregation-caused quenching phenomenon that is observed for many of these species in the solid-state and surpass the solubility restrictions usually visible in these cases [26]. Few polymer nanoparticles doped with luminescent coordination compounds have been reported to date to be potentially valuable for biomedical applications. Herein, we have performed a proof of concept of co-encapsulation of both the B(III) compounds as the emissive dye and the coumarin 6 as the hydrophobic drug. The new nanocomposites behave as efficient drug delivery materials.

## 2. Materials and Methods

### 2.1. Reagents and Materials

The β-diketonate boron(III) compounds were synthesized according to the procedures previously established by us [22]. Coumarin-6, polymethylmethacrylate (PMMA), polyvinylpyrrolidone (PVP) and poly(styrene-butadiene-styrene) (SBS) were purchased from Sigma-Aldrich (St. Louis, MO, USA). Solvents were purchased from PanReac (Castellar del Vallès, Barcelona, Spain) and used without further purification. Ultrapure deionized water was used for the synthesis of particles.

### 2.2. Methods

The UV–Vis absorption, fluorescence emission and excitation spectra were obtained using a Jasco V-650 spectrophotometer (JASCO International, Tokyo, Japan) and a Horiba-Jobin-Yvon Fluoromax-4 spectrofluorimeter (Horiba Scientific, Palaiseau, France) at Proteomass-Bioscope facilities. All the spectra were recorded at room temperature (298 K) in an air atmosphere by exciting at 345 nm.

Dynamic light scattering (DLS) studies were carried out by using a Malvern Zetasizer Nano series (Worcestershire, WR14 1XZ, Malvern, UK) from PROTEOMASS-BIOSCOPE facilities. Glass and dip cells were used for the record of size distributions and *ζ*-potential, respectively, at a scattering angle of 90° and 20 °C. A stabilization of 5 min was required during DLS studies at variable temperatures before reaching the corresponding temperature.

The morphology of the particles was studied by using a JEOL JEM 1010 transmission electron microscope at CACTI, University of Vigo (Vigo, Spain). After dropping five μL of the corresponding aqueous dispersions over a copper grid coated with a carbon film, water was allowed to evaporate. The images were obtained by operating at 100 kV. ImageJ software was used for the analysis of data (Image 1.51 h, Wayne Rasband, National Institutes of Health, Bethesda, MD, USA) 

### 2.3. Synthesis of Polymer Particles Doped with the B(III) Compounds

A solution of the selected compound BF12, BF14 or BF16 (0.5 mg), and the corresponding polymer matrix PMMA, SBS or PVP (50 mg) in 1 mL of tetrahydrofuran (THF) was prepared and heated at 50 °C for 3 min to allow solubilization. When the solution was cooling back again to room temperature, an aliquot of 0.6 mL was quickly added over 2.4 mL of deionized water, maintaining a magnetic stirring at 400 rpm for 5 min. The resulting mixture was stored at 20 °C without contact with light for 24 h for stabilization of oil-in-water droplets. The final particles were obtained after evaporation of THF at room temperature for 24 h. Before use, a filtration step using glass membranes with a pore size of 10–15 μm was performed to remove potential non-encapsulated B(III) and polymer precipitates.

### 2.4. Preparation of Coumarin-6 Loaded Particles

A coumarin 6 (C6) solution was previously prepared in 10 mL of THF (10^−3^ M). Then, an aliquot of 1 mL was used to solve 0.5 mg of BF14 and 50 mg of PMMA, heating at 50 °C for 3 min to allow solubilization. After cooling back to room temperature, 0.6 mL of the resulting mixture was quickly added to 2.4 mL of deionized water under magnetic stirring at 400 rpm for 5 min. To promote the stabilization of the oil-in-water droplets, the solution was stored at 20 °C without contact with light for 24 h. Slow evaporation of THF was performed at room temperature for 24 h without stirring, resulting in a dispersion of the proper C6-loaded particles. The filtration step using glass membranes with a pore size of 10–15 μm was used for the aqueous solutions to remove any precipitate of BF14, PMMA and C6.

## 3. Results

### 3.1. Preparation and Characterization of Micro- and Nanoparticles

**Figure 2:**
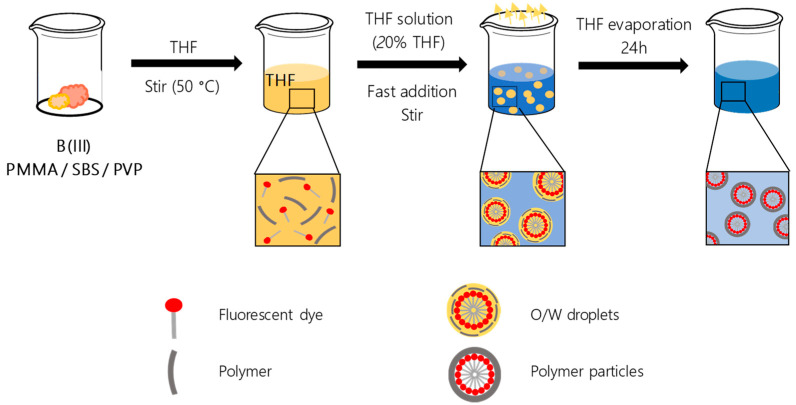
Synthesis of the polymer particles doped with the B(III) compounds.

The polymer particles encapsulating the B(III) compounds were prepared via the reprecipitation method by using THF as the oil phase and water (Figure 2) [17,27]. Oil-in-water (O/W) droplets containing the polymer matrix and the selected compound are formed by fast addition of both materials solved in THF over water. After stabilizing the colloidal solution for 24 h, THF is allowed to evaporate slowly for 24 h, and the final polymer particles dispersed in water are obtained.

**Figure 3:**
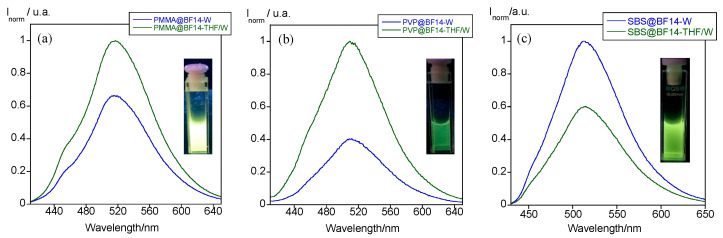
Emission spectra of (**a**) PMMA@BF14, (**b**) PVP@BF14, and (**c**) SBS@BF14 inside the O/W droplets (THF/W) and dispersed in water (W). The inset displays an image of the luminescence emission of these particles in water under UV light (λ_exc_ = 365 nm).

**Figure 4:**
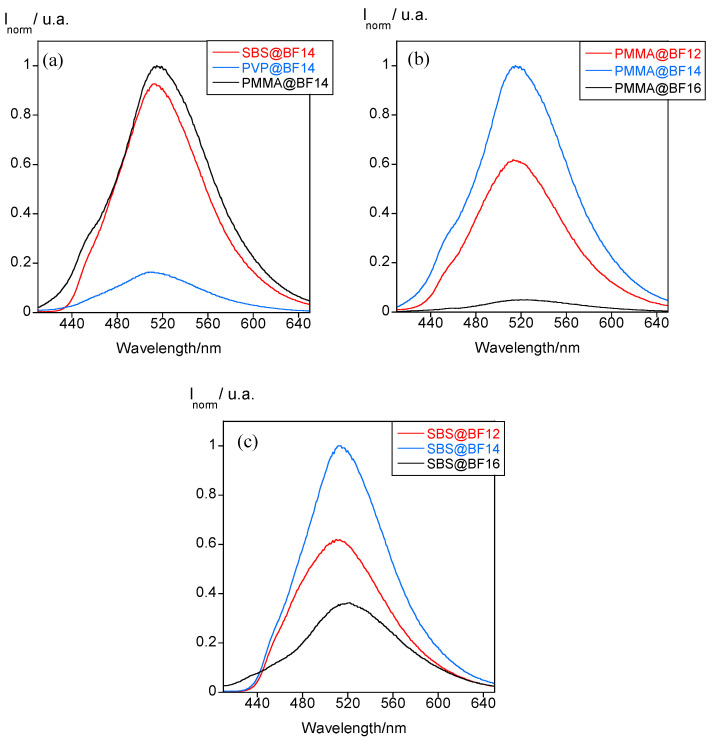
(**a**) Emission spectra of SBS, PVP and PMMA particles doped with BF14. (**b**,**c**) Emission spectra of PMMA and SBS particles doped with all the B(III) compounds.

The B(III)-doped polymer particles show a greenish luminescence in the O/W droplets and the water suspension. Although THF evaporation does not cause any shift in the emission band, its intensity notably changes in the three new materials. In the case of PMMA@BF14 and PVP@BF14, a decrease of the fluorescence was observed after the evaporation process, whereas an opposite behavior is found for SBS@BF14 (Figure 3a–c). No precipitation was detected in any case, which indicates that these variations should be related to the potential interactions that the polymer matrix establishes with the molecules of the compound inside the particle. The presence of aromatic rings in the structure of SBS could originate a more efficient π-stacking with the B(III) compound, enhancing its fluorescence emission in the absence of THF. The best polymer matrixes that enhance the boron compounds’ fluorescence are PMMA and SBS, as demonstrated in Figure 4a. The PVP matrix is not a good candidate to fabricate these polymer micro and nanoparticles since the natural emission of BF14 is quenched. Thus, we selected PMMA and SBS as the matrixes to study the effect of the alkyl chain length. As expected, the solubility of the B(III) compound bearing 16 carbon atoms is lower than that of the analogous compound with shorter alkyl chains, so that the amount of encapsulated compound is lower and, therefore, also its luminescence emission (Figure 4b,c).

Interestingly, dispersion of B(III) compounds inside the polymer matrix is effective, and it avoids the aggregation-induced fluorescence quenching effect commonly observed for this type of fluorophores. The maximum amount of BF14 entrapped into PMMA was calculated to be only just 4.93 µg (3.39 µM); these particles, in turn, are the most emissive. The low concentration allows maintaining, on the one hand, the photophysical properties of BF14 in the aqueous dispersion (avoiding aggregation-caused quenching) and, on the other hand, the morphology, size and colloidal stability of the B(III)-doped particles.

**Figure 5:**
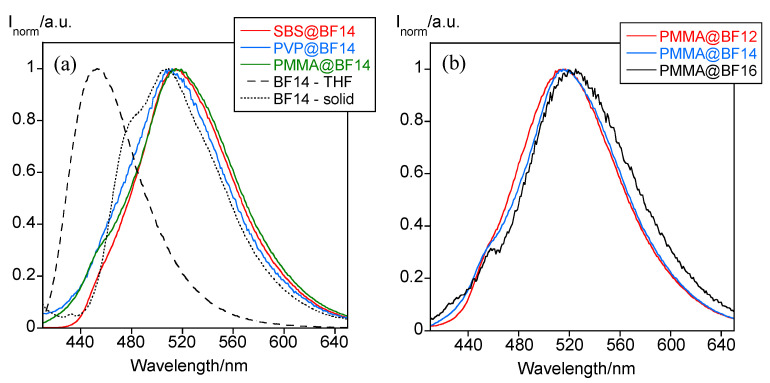
(**a**) Normalized emission spectra of the polymer particles doped with BF14. The emission spectra of BF14 in THF solution and in the solid-state are also shown for comparative purposes. (**b**) Normalized emission spectra of PMMA particles doped with all the B(III) compounds studied.

Regarding the position of the emission band, it appears centered at ca. 509–515 nm regardless of the polymer matrix used (Figure 5a), and the alkyl chain length seems to have no significant effect on it neither (Figure 5b). However, the emission maximum is 62 and 6 nm red-shifted with respect to than found for the free compound in THF and in the solid state, respectively (Figure 5a), which can be associated with the existence of interligand charge transfers as a result of the formation of intermolecular interactions [22].

DLS measurements established the hydrodynamic diameter and ζ-potential of the new materials. Table 1 collects the DLS data for all samples analyzed.

**Figure 6:**
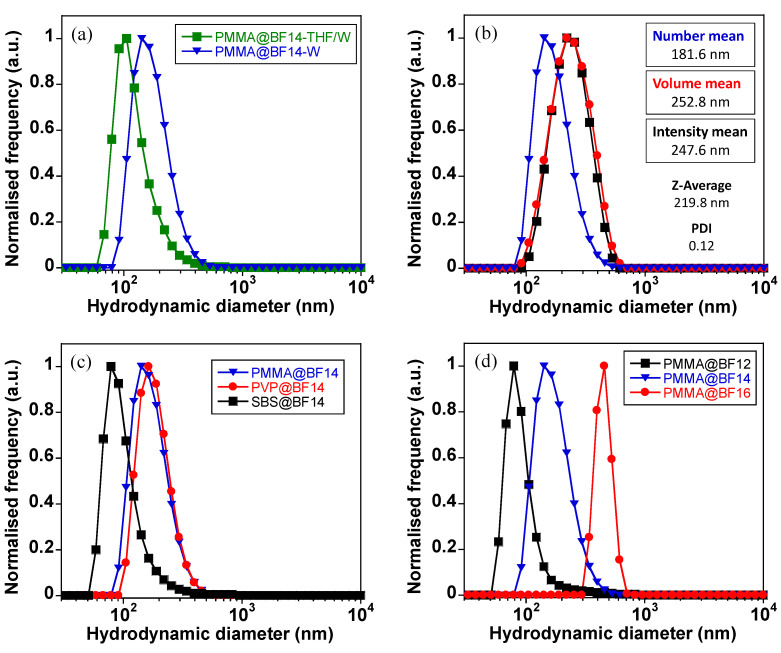
DLS data. (**a**) Number size distribution recorded for PMMA@BF14 in the O/W droplets and after evaporation of THF. (**b**) DLS spectra showing the number, volume and intensity size distributions of PMMA@BF14 in the colloidal suspension. (**c**) Number size distribution of PMMA, PVP and SBS particles doped with BF14. (**d**) Number size distribution of PMMA particles doped with BF12, BF14 and BF16.

The O/W droplets act well as a template, and it is observed that only slight variations in the hydrodynamic diameter of the final particles are caused during the THF evaporation process (Figure 6a). The number, volume, and intensity plots of polymer particles show a unique size distribution with slight discrepancy among them, the best results being found for PMMA@BF14, which displays a low polydispersity index (PDI) value of 0.12 (Figure 6b). It is interesting to remark that the average size varies as a function of the polymer matrix and the alkyl chain length. By comparing the average hydrodynamic diameter of all the polymer particles doped with BF14, note that the polymer particles have similar sizes of about 200 nm (see Table 1). This result demonstrates that the polymer matrix does not affect drastically to the particle size (Figure 6c), the most notorious difference being found when the alkyl chain length (n) of the B(III) compound is analyzed. The PMMA particles obtained from the B(III) compound with *n* = 16 carbon atoms have an average hydrodynamic diameter of 1120 nm, which highly contrasts with that found in PMMA particles doped with shorter alkyl chains (below 300 nm; see Table 1). This fact suggests that the increase of the chain length from 14 to 16 carbon atoms plays a key role in how molecules are packed inside PMMA, and it strongly depends on the solubility of these systems in THF. Whereas BF12 and BF14 show a similar solubility in THF and allow obtaining particles with a non-significant difference in size, the solubility of BF16 is poor, which hinders the assembly with PMMA inside the oil-in-water droplets and therefore, polymer particles notably grow in size (Figure 6d).

**Figure 7:**
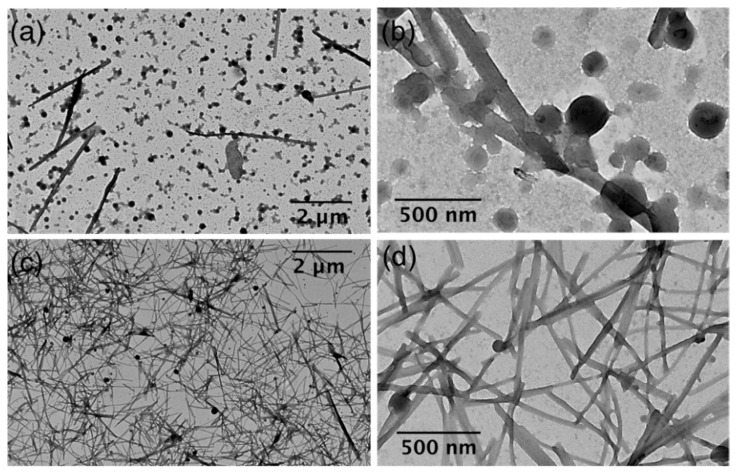
Transmission electron microscopy (TEM) images of (**a**,**b**) PMMA@BF4, and (**c**,**d**) SBS@BF14.

**Figure 8:**
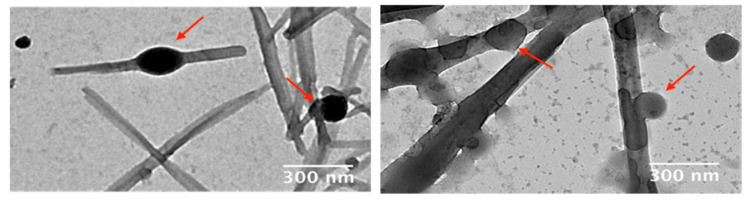
TEM images showing examples of spherical nanoparticles reorganized in fibers.

**Figure 9:**
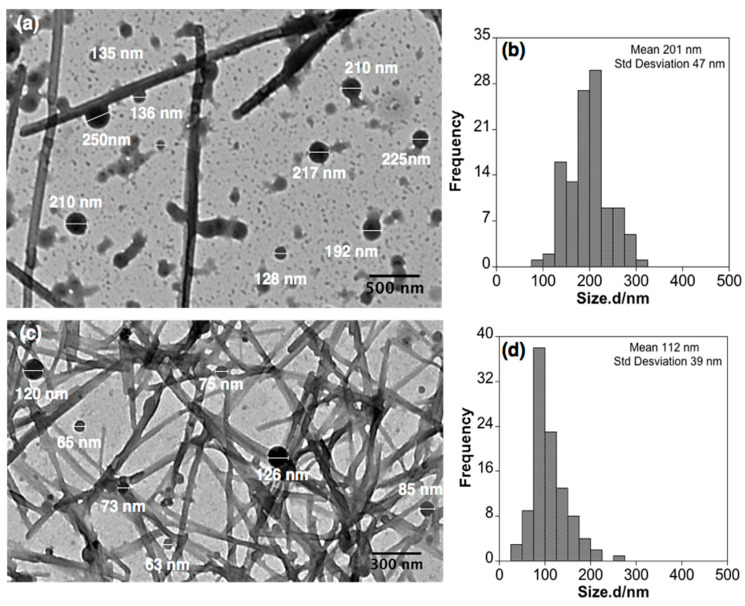
TEM images showing examples of spherical nanoparticles considered in the histogram for PMMA@BF14 (**a**,**b**) and SBS@BF14 (**c**,**d**).

To further investigate the nanostructure of the B(III)-doped materials, transmission electron microscopy (TEM) analyses were performed. Surprisingly, in all samples investigated at low magnification, we observe the presence of a fiber-like material, together with a remaining population of spherical particles (see Figure 7a,c). We suggest that this unexpected reorganization from particles to fibers (spanning sizes greater than 2 µm) should be associated with the drying effect of colloidal solutions for microscopy analysis. On the one hand, DLS measurements for the colloidal suspension of PMMA@BF14 did not show significant changes for four weeks, which indicates that the particles dispersed in water are stable for at least a month. Furthermore, on the other hand, as shown in Figure 7b,d and Figure 8, TEM images at higher magnification reveal several nanoparticles that apparently show intermediates in the reorganization process towards fiber-like structures. Considering the spherical nanoparticles, the statistical analysis shows a concordance with the light scattering data, obtaining a mean size of 201 nm and 112 nm for PMMA@BF14 and SBS@BF14, respectively (see Figure 9).

### 3.2. Temperature-Dependent Emission Properties

**Figure 10:**
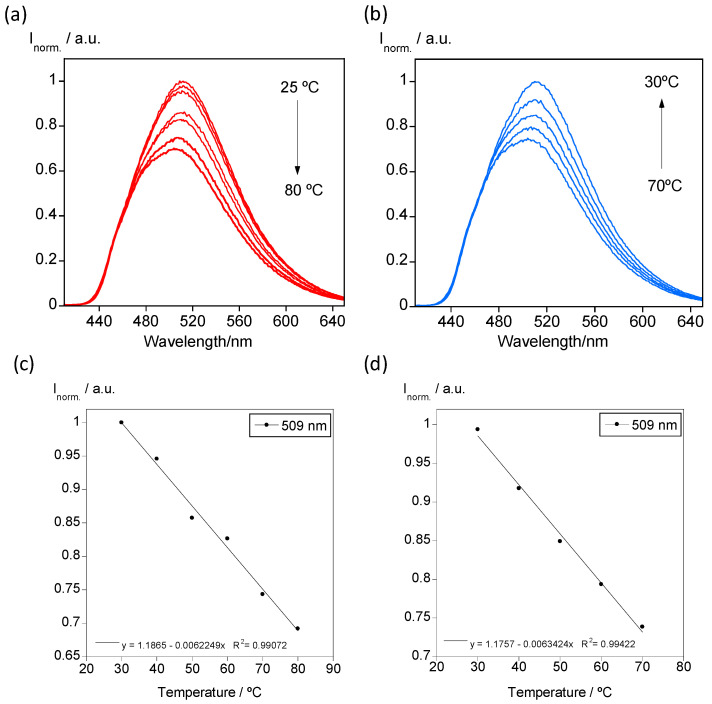
(**a**,**b**) Temperature-dependent emission spectra of SBS@BF12 upon heating and cooling. (**c**,**d**) Calibration curve obtained by plotting the maximum emission data recorded at 509 nm as a function of temperature.

The luminescence emission of PMMA, PVP and SBS polymer particles doped with BF14 was studied at variable temperature from 20 °C to 80 °C. In Figure 10a, it is shown the temperature-dependent emission spectra recorded for SBS@BF12 as a representative example of all the colloidal suspensions. As expected, the increase in temperature produces a quenching of the emission, although the colloidal suspension emits greenish light at high temperatures. When the sample is cooling back to room temperature, the emission is restored, and the thermal behavior shows high reversibility (Figure 10b). Moreover, the emission intensity as a function of temperature presents linearity, as shown in Figure 10c,d. This result is an important characteristic that demonstrates the potential use of these systems in nanothermometry.

### 3.3. Hydrophobic Drug Entrapment into B(III)-Doped Microparticles

**Figure 11:**
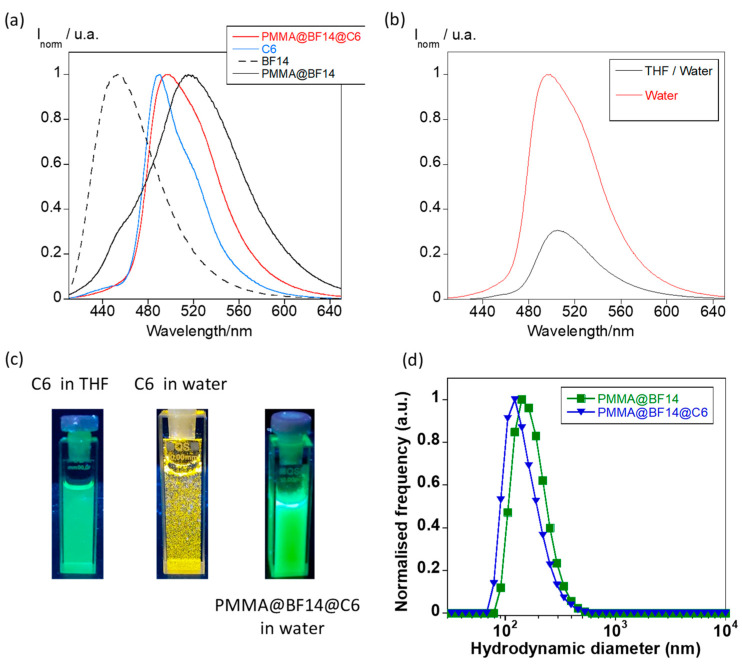
(**a**) Normalized emission spectra for C6 and BF14 in THF, and PMMA@BF14 and PMMA@BF14@C6 in water. (**b**) Emission spectra of the O/W droplets containing PMMA@BF12@C6 in THF/water, and final PMMA@BF12@C6 particles dispersed in water. (**c**) Images showing the fluorescence emission of C6 in THF solution, the precipitates of C6 in water, and the bright emission of PMMA@BF14@C6 dispersed in water. All images were taken under UV light (λ_exc_ = 365 nm). (**d**) DLS spectra were recorded for PMMA@BF14@C6 (number distribution). The number size distribution of PMMA@BF14 is also plotted for comparison.

Over the last years, the entrapment of hydrophobic drugs into polymer particles has attracted significant attention for application in biomedicine [28]. In this context, we were interested in exploring the entrapment ability of the B(III)-doped particles described in this work. Keeping in mind that they also exhibit luminescence properties, the new materials could concomitantly behave as drug transports and bioimage probes. For these studies, C6 was used as a model of hydrophobic drug [29,30]. It emits a bright greenish fluorescence in THF (λ_em_ = 489 nm), but it is not soluble in water as evidenced by exposure to the mixture under UV light (Figure 11a–c). This photophysical behavior makes it a candidate suitable to study the entrapment process via fluorescence spectroscopy.

PMMA@BF14@C6 particles were prepared from a THF solution of C6 and BF14 following the reprecipitation method used to synthesize the B(III)-doped particles (see the experimental section for more details). Interestingly, the new PMMA particles show a bright greenish emission with the maximum centered at 497 nm and a shoulder at 518 nm, consistent with the contributions of both C6 and BF14, confirming the entrapment of C6 inside the polymer matrix (Figure 11a). Comparative analysis of the emission spectra of the initial O/W droplets containing the PMMA@BF14@C6 and the final particles obtained evidence of drastic changes. The emission intensity is enhanced when THF is evaporated (Figure 11b). This fact can be understood keeping in mind that C6 and BF14 precipitate inside the polymer matrix during THF evaporation, and therefore, their emission properties are measured in the solid state although particles are dispersed in water.

DLS spectra for PMMA@BF14@C6 are shown in Figure 11d. It can be observed that the entrapment of C6 does not drastically disturb the size distribution of the particles, which show an average size of 152 nm and a PDI value of 0.21. Likewise, the zeta potential was found to be about −38.9 mV, a value similar to that obtained for PMMA@BF14.

## 4. Discussion

As stated above, compounds BF12, BF14, and BF16 were described in a previous work, and their photophysical behavior was studied [22]. Since their luminescence nature, they were used as dopants to fabricate highly fluorescent PMMA, PVP, and SBS thin films. Unfortunately, these B(III) compounds are non-soluble in water, which significantly limits their potential applications in biomedicine. Herein, we have used the reprecipitation method to achieve the encapsulation of these species in a polymer matrix at the nanoscale and thus maintain the emission properties in water.

Interestingly, the polymer micro and nanoparticles show the emission maximum centered at around 509–515 nm. This value is in agreement with that found for the B(III) compounds in the solid state (λ = 520, 508 and 497 nm for BF12, BF14 and BF16, respectively), whereas it is blue-shifted with respect to that found in solution (λ = 552 nm for the three compounds). This can be understood because the B(III) compounds entrapped into the polymer particles precipitate during THF evaporation and are entirely isolated from the aqueous medium.

On the other hand, by comparing the emission band of the dyes inside the polymer thin films and nanoparticles, it is observed that the effect of the polymer matrix in thin films (λ = 460, 520 and 470 nm for PMMA, PVP and SBS, respectively) is not maintained at the nanoscale, in which the emission maximum appears at 509–515 nm regardless the polymer used. These differences are mainly due to the dispositions that molecules adopt in the resulting materials. Results seem to indicate that intermolecular interactions between the dyes and polymer matrixes drive the assembly of the B(III) compounds in thin films, whereas for nanoparticles, the dye molecules adopt an assembly imposed by the oil-in-water droplets and the subsequent THF evaporation. In fact, temperature-dependent studies evidenced notable changes in the emission properties of thin films that could be associated with a reorganization of the dyes via intermolecular interactions, especially for SBS films due to its elasticity; by contrast, no changes were attributed to the molecular assembly were observed in the particles.

It has also been established that the encapsulation of the B(III) compounds does not affect the morphology, size, or colloidal stability of the polymer particles. Similar PMMA-co-MMA particles doped with quantum dots have been reported in the literature to be useful for cellular and tissue bioimage, demonstrating the biocompatibility of PMMA derivatives [17]. Encapsulation of coordination compounds has also been described by using PS or polymer/lipid matrixes [11,18], achieving average size, PDI and zeta potential values similar to those reported by us for the B(III) compounds, in all cases below 250–300 nm, 0.2–0.3 and 20–30 mV, respectively. Interestingly, for the particular case of diruthenium-doped particles, the co-entrapment of ibuprofen and naproxen metallodrugs enhanced the cytotoxicity in breast and prostate cancer cells. In this context, we have demonstrated through a proof of concept that co-encapsulation of a hydrophobic drug model, C6, is possible, the polymer B(III)-doped particles being potential candidates for use as drug nanocarriers.

## 5. Conclusions

In this work, different PMMA, PVP, and SBS polymer micro- and nanoparticles doped with a series of hydrophobic β-diketonate B(III) compounds as emissive materials have been prepared. The entrapment of compounds inside the polymer matrix in these nanoparticles was achieved, forming oil-in-water droplets via the reprecipitation method using THF as the oil phase, which originates remarkable luminescent materials. The new micro and nanomaterials synthesized show an average size ranging between 160 and 1120 nm, high colloidal and thermal stability, and luminescence properties during at least one month. Drastic changes associated with interligand charge transfer effects in the B(III) compounds are observed regarding the luminescence response. This confirms the existence of intermolecular interactions between the compounds and the different polymer matrixes used. Finally, we explored the B(III)-doped polymer particles as drug inclusion systems by encapsulating a hydrophobic drug model, the coumarin 6, which is an excellent example of drug transport and bioimaging nanoprobes. Further studies with different cell lines and drugs are in progress.

## Figures and Tables

**Table 1:** Dynamic light scattering data for the obtained particles.

Particles	Z-Average (nm)	PDI	ζ-Potential (mV)
PMMA@BF12	284.4	0.39	−43.2
PMMA@BF14	219.8	0.12	−40.3
PMMA@BF16	1120.0	0.76	−23.4
PVP@BF14	231.0	0.10	−40.0
SBS@BF12	380.1	0.33	−41.3
SBS@BF14	244.2	0.38	−39.4
SBS@BF16	190.0	0.21	−60.4

## Data Availability

Not applicable.
